# Topological vacuum bubbles by anyon braiding

**DOI:** 10.1038/ncomms11131

**Published:** 2016-03-31

**Authors:** Cheolhee Han, Jinhong Park, Yuval Gefen, H.-S. Sim

**Affiliations:** 1 Department of Physics, Korea Advanced Institute of Science and Technology, 291, Daehak-ro, Yuseong-gu, Daejeon 34141, Korea; 2 Department of Condensed Matter Physics, Weizmann Institute of Science, Rehovot 76100, Israel

## Abstract

According to a basic rule of fermionic and bosonic many-body physics, known as the linked cluster theorem, physical observables are not affected by vacuum bubbles, which represent virtual particles created from vacuum and self-annihilating without interacting with real particles. Here we show that this conventional knowledge must be revised for anyons, quasiparticles that obey fractional exchange statistics intermediate between fermions and bosons. We find that a certain class of vacuum bubbles of Abelian anyons does affect physical observables. They represent virtually excited anyons that wind around real anyonic excitations. These topological bubbles result in a temperature-dependent phase shift of Fabry–Perot interference patterns in the fractional quantum Hall regime accessible in current experiments, thus providing a tool for direct and unambiguous observation of elusive fractional statistics.

## Abstract (web summary)

Quantum vacuum fluctuations can create pairs of virtual particles that annihilate each other. Here, the authors show that the conventional theory that these particles have no measurable effect on real particles does not apply to anyons, exotic quasiparticles that are intermediate between fermions and bosons.

## Graphical abstract

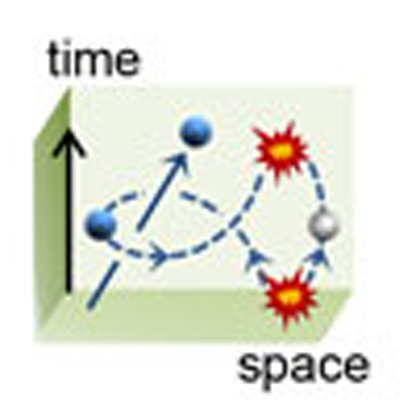

When two identical particles adiabatically exchange their positions **r**_i=1,2_, their final state 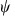 (up to dynamical phase) is related to the initial one through an exchange statistics phase θ*,


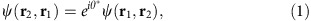


with θ*=0 (*π*) for bosons (fermions)^1^.

Anyons^2^,^3^,^4^ are quasiparticles in two dimensions, not belonging to the two classes of elementary particles, bosons and fermions. Abelian anyons appear in the fractional quantum Hall (FQH) system of filling factor *ν*=1/(2*n*+1), *n*=1,2,⋯. They carry a fraction e*=*νe* of the electron charge *e* and obey fractional exchange statistics, satisfying equation (1) with θ*=±*πν*. Two anyons gain a phase ±2*πν* from a braiding, whereby one winds around the other; ± depends on the winding direction. Although fractional charges have been detected^5^,^6^,^7^,^8^, experimental measurement of statistics phase *πν* has been so far elusive. Existing theoretical proposals for the measurement involve quantities inaccessible in current experiments or suffer from unintended change of a proposed setup with external parameters^9^,^10^,^11^,^12^,^13^,^14^,^15^,^16^,^17^,^18^.

In many-body quantum theory^1^, Feynman diagrams are used to compute the expectation value of observables. This approach invokes vacuum bubble diagrams, which describe virtual particles excited from vacuum and self-annihilating without interacting with real particles. According to the linked cluster theorem^1^, each diagram possessing vacuum bubbles comes with, hence is exactly cancelled by, a partner diagram of the same magnitude but of the opposite sign. Consequently, vacuum bubbles do not contribute to physical observables.

In the following, we demonstrate that this common wisdom has to be revised for anyons: a certain class of vacuum bubbles of Abelian anyons does affect observables. These virtual particles, which we call topological vacuum bubbles, wind around a real anyonic excitation, gaining the braiding phase ±2*πν*. We propose a realistic setup for detecting them and θ*=*πν*.

## Results

### Topological vacuum bubble

**Figure 1:**
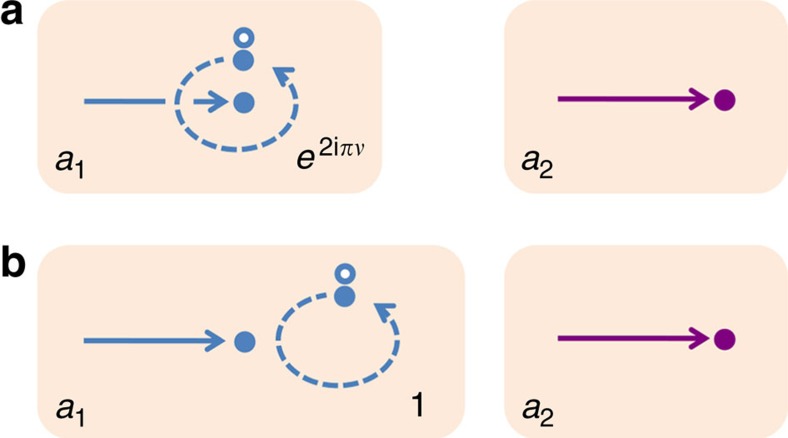
Topological vacuum bubble. Feynman diagrams for interference involving a real particle and a virtual particle–hole excitation from vacuum. Full (empty) circles represent particles (holes). Solid (dashed) lines denote propagations of real (virtual) particles. (**a**) Diagram for the interference 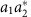 of two processes: (*a*_1_, blue) A real particle propagates, a virtual particle–hole pair is excited, then the pair self-annihilates after the virtual particle winds around the real one. (*a*_2_, magenta) A real particle propagates. The entire virtual process constitutes a vacuum bubble. For anyons, the bubble gains a topological braiding phase 2*πν* from the winding. (**b**) Partner diagram of **a**. Here a virtual particle, constituting another bubble, does not encircle a real one and hence gains no braiding phase. The diagrams in **a** and **b** contribute to observables for anyons, while they do not for bosons and fermions.

We illustrate topological vacuum bubbles. In Fig. 1a, a Feynman diagram represents interference 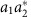 between processes *a*_1_ and *a*_2_ of propagation of a real particle. In *a*_1_, a virtual particle-hole pair is excited then self-annihilates after the virtual particle winds around the real particle, forming a vacuum bubble, while it is not excited in *a*_2_. The winding results in a braiding phase 2*πν* and an Aharonov–Bohm phase 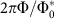 from the magnetic flux Φ enclosed by the winding path, contributing to the interference signal as 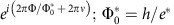 is the anyon flux quantum^9^,^19^.

The limiting cases of bosons (*ν*=0) and fermions (*ν*=1) imply that this bubble diagram appears together with, and is cancelled by, a partner diagram in Fig. 1b. The partner diagram has a bubble not encircling the real particle and involves only 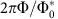. The two diagrams (and their complex conjugates) yield


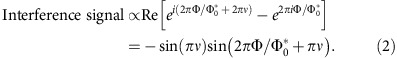


For bosons and fermions, the two diagrams fully cancel each other with sin(*πν*)=0 in agreement with the linked cluster theorem; hence, the signal disappears. By contrast, for anyons they cancel only partially, producing the non-vanishing interference in an observable, and are topological as the braiding phase is involved.

### Interferometer setup

**Figure 2:**
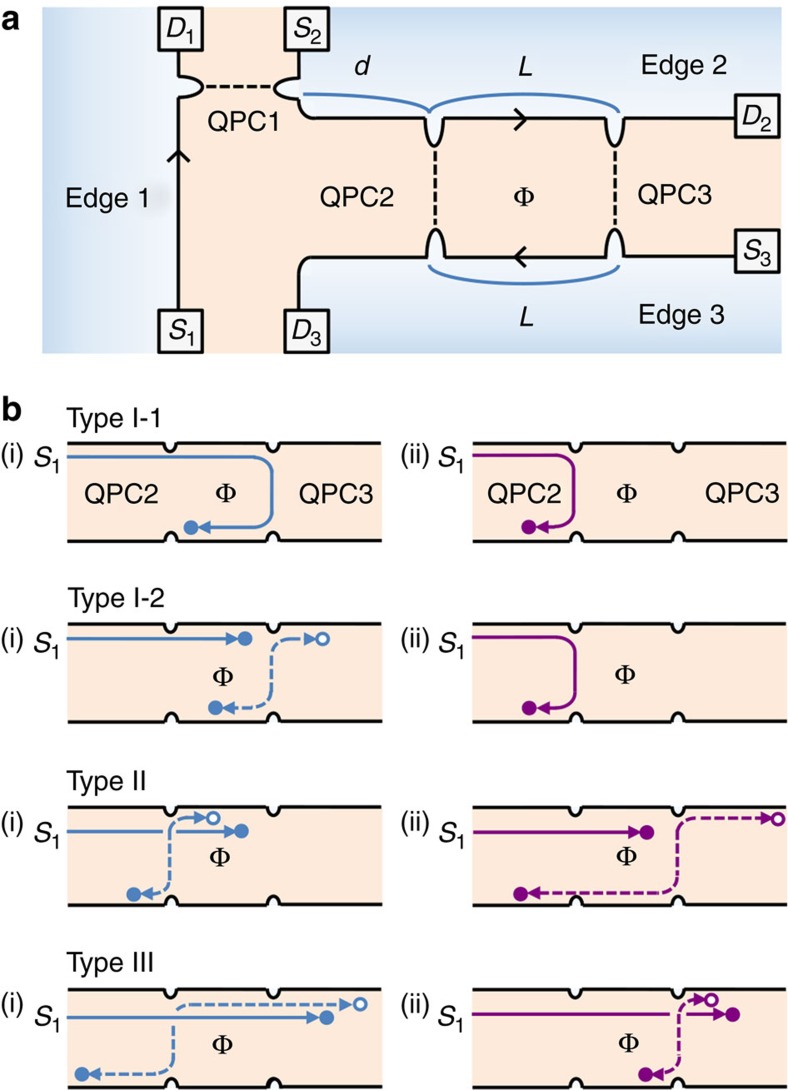
Interferometry for detecting topological vacuum bubbles. (**a**) In the setup, anyons move (see arrows) along FQH edge channel *i*=1,2,3 that connects source *S*_*i*_ and drain *D*_*i*_, and jump (dashed) between the channels via tunnelling at QPCs. The loop defined by Edges *i*=2,3, QPC2 and QPC3 encloses magnetic flux Φ, forming a Fabry–Perot interferometer. Distance between QPC2 and QPC3 (QPC1) is *L* (*d*). (**b**) Two interfering paths (i) and (ii) of each main interference process at 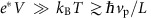. Following Fig. 1, filled (empty) circles represent particle-like (hole-like) anyons and solid (dashed) lines denote propagation of an anyon injected from *S*_1_ (anyon pair excitation at QPCs). Type II and III processes involve a topological vacuum bubble.

In Fig. 2a, we propose a minimal setup for observing topological vacuum bubbles. It is a Fabry–Perot interferometer^9^,^17^,^20^,^21^,^22^,^23^ in the *ν*=1/(2*n*+1) FQH regime, coupled to an additional edge channel (Edge 1) via a quantum point contact (QPC1). At QPC*i*, there occurs tunnelling of a single anyon (rather than anyon bunching), fulfilled^24^ with 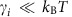; *γ*_*i*_ is the tunnelling strength and *T* is the temperature. Gate voltage *V*_G_ is applied, to change the interferometer loop enclosing Aharonov–Bohm flux Φ. The interference part 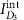 of charge current at drain *D*_3_ is measured with bias voltage *V* applied to source *S*_1_; the other *S*_*i*_'s and *D*_*i*_'s are grounded. Together with ‘virtual' (thermal) anyon excitations in the interferometer, a voltage-biased ‘real' anyon, dilutely injected at QPC1 from Edge 1 to the interferometer, forms topological vacuum bubbles, as shown below. The bubbles contribute to 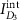 at the leading order 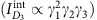 in QPC tunnelling, as Edges 2 and 3 are unbiased. It is noteworthy that in the setups previously studied^9^,^10^,^11^,^12^,^13^,^14^,^15^,^16^,^17^,^18^, topological bubbles do not contribute to current at the leading order.

We consider the regime of 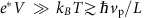, where the size *L*_*V*_≡*ℏv*_p_/(e*V) of the dilutely injected anyons is much smaller than interferometer size *L* and the injection of hole-like anyons at QPC1 is ignored; *v*_p_ is anyon velocity along the edges and e*V should be much smaller than the FQH energy gap. Because of the dilute injection and 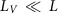, anyon braiding is well defined in the interferometer. As shown below, the dependence of 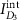 on Φ or on *V*_G_ provides a clear signature of the topological bubbles, consequently, θ*=*πν* in both of the pure Aharonov–Bohm regime (where Coulomb interaction of the edge channels with bulk anyons localized inside the interferometer loop is negligible) and the Coulomb-dominated regime (where the interaction is strong)^22^,^25^. Below, we first ignore bulk anyons.

### Interference current

Employing the chiral Luttinger liquid theory^26^,^27^ for FQH edges and Keldysh Green's functions^10^,^12^, we compute 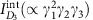 at the leading order in *γ*. There are four types of the processes mainly contributing to 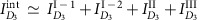; see Fig. 2b. For 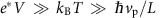, we obtain the analytical expression of 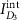: The interference current contributed by Type I-1 processes is 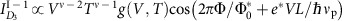, which by Type I-2 is 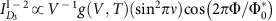, and those by Type II and III are


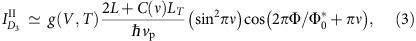



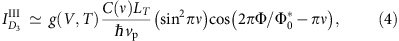


where 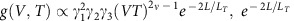 is a thermal suppression factor, thermal length *L*_*T*_≡*ℏv*_p_/(*πνk*_B_*T*) and 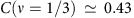; see Methods and the Supplementary Note 3.

Type I-1 processes describe interference between two paths of an anyon moving from *S*_1_ to *D*_3_ via (i) QPC2 and (ii) QPC3, respectively. They were previously studied^9^.

In Type I-2, an anyon injected from *S*_1_ interferes with a particle-like anyon excited at a QPC or annihilates a hole-like anyon; particle-like and hole-like anyons are pairwise excited thermally at QPCs. For example, consider the two following interfering histories: (i) an anyon is injected from *S*_1_ to *D*_2_, an anyon pair is excited at QPC3 and then the particle-like (hole-like) anyon of the pair moves to *D*_3_ (*D*_2_). (ii) An anyon is injected from *S*_1_ to *D*_3_ via QPC2 without any excitations. The hole-like anyon annihilates the injected anyon on Edge 2 in history (i) and the particle-like anyon of (i) interferes with the injected anyon of (ii) on Edge 3. The sum of such interference processes yields 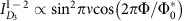. The sin^2^*πν* factor appears, because relative locations of anyons on Edge 2 or 3 differ between the processes, leading to an exchange phase ±*πν*, and because a process with an excitation (of a particle-like anyon moving to *D*_2_ and a hole-like one to *D*_3_) yields charge current in the opposite direction to another with its particle-hole conjugated excitation (of a particle to *D*_3_ and a hole to *D*_2_).

In Types II and III, a real anyon injected from *S*_1_ moves to *D*_2_ and a virtual anyon pair excited at QPC2 interferes with another at QPC3. The interference path effectively encloses the real anyon, forming a topological vacuum bubble (*cf*. Fig. 1). In Type II, when the real anyon is located on Edge 2 between QPC2 and QPC3, a virtual pair is excited at QPC2 in history (i) and at QPC3 in history (ii). Next, the hole-like (particle-like) anyon of each pair moves, for example, to *D*_2_ (*D*_3_). The interference of the two histories corresponds to the winding of a virtual anyon around the real one and Φ, forming a topological bubble with interference phase 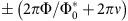; ±depends on whether the hole-like anyon moves to *D*_2_ or *D*_3_. In the interference, the winding of a virtual anyon around the real one effectively occurs through the exchanges of the positions of the anyons in each of Edges 2 and 3, as relative locations of anyons on Edges 2 and 3 differ between (i) and (ii) (see Supplementary Fig. 2 and Supplementary Note 6). This interference is accompanied by a partner process. The latter has a bubble that winds around Φ (gaining phase 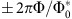), but not around a real anyon. The two partner processes partially cancel each other, yielding 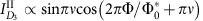; the remaining sin *πν* factor in equations (3) and (4) has a similar origin to the sin^2^ *πν* factor of 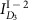.

In Type III, the two interfering histories are as follows: (i) a virtual pair is excited at QPC2 before a real anyon injected from *S*_1_ arrives at QPC2 and (ii) another pair is excited at QPC3 after the real one arrives at QPC3. The ensuing chronological sequence on Edge 2 is opposite to Type II: an anyon excited at QPC2 arrives at QPC3; the real one arrives at QPC3; a pair is excited at QPC3. The resulting topological bubble effectively winds around the real anyon in the direction opposite to its winding around Φ, yielding a phase 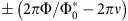. Partial cancellation of the bubble and its partner leads to 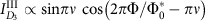. The factor 2*L*+*CL*_*T*_ of 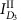 (*CL*_*T*_ in 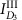) in equation (3) (equation (4)) comes from the time window compatible with the chronological sequence on Edge 2.

Type II and III processes of topological bubbles do not affect any observables at *ν*=1 (fermions), due to full cancellation between partner bubbles (the linked cluster theorem). They are distinct from I-2. I-2 processes produce, for example, non-vanishing current noise 
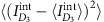
 at *ν*=1, as the particle-hole conjugated excitations (mentioned before) equally contribute to the noise (although the contributions of the conjugations to 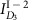 cancel each other, leading to 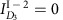).

In the more general regime of 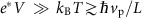, we employ the parametrization


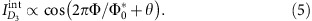


The phase *θ* is determined by competition between the various contributions to 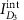 and contains information about statistics phase *πν*. At 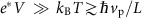, 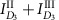 is much larger than 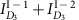 and dominates 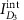, because the interfering anyon of Types I-1 and I-2 is voltage biased and has width *L*_*V*_∝*V*^−1^ much narrower than the thermal anyon excitations (whose width *L*_*T*_∝*T*^−1^) of II and III, showing much weaker interference. From 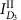 and 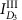, we find


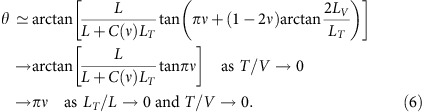


The arctan 2*L*_*V*_/*L*_*T*_ term represents an error in the braiding phase 2*πν* of the topological bubbles. It occurs when the size *L*_*V*_ of a real anyon is not sufficiently smaller than the winding radius of a virtual anyon around the real one. It is negligible at 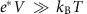, as the radius is effectively large when 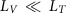; those corresponding to the error are ignored in equations (3) and (4), and are shown in Supplementary Note 3. For 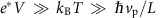, 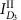 dominates 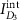 and *θ*→*πν*. Remarkably, *θ* depends on *T*, contrary to common practice in electron interferometry^28^.

### Coulomb-dominated regime

In experimental situations of a Fabry–Perot interferometer in the FQH regime, it is expected that there exist bulk anyons localized inside the interferometer loop. There are two regimes of Fabry–Perot interferometers, the pure Aharonov–Bohm regime and the Coulomb-dominated regime. In the former regime, Coulomb interaction between the bulk anyons and the edge of the interferometer is negligible, whereas it is crucial in the latter^25^. The Fabry–Perot interferometers of recent experiments^17^,^20^,^21^,^22^,^23^ in the FQH regime are in the Coulomb-dominated regime. Below we compute the interference current 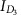 in the presence of the Coulomb interaction and show that equation (6) is applicable to both of the pure Aharonov–Bohm regime and the Coulomb-dominated regime.

**Figure 3:**
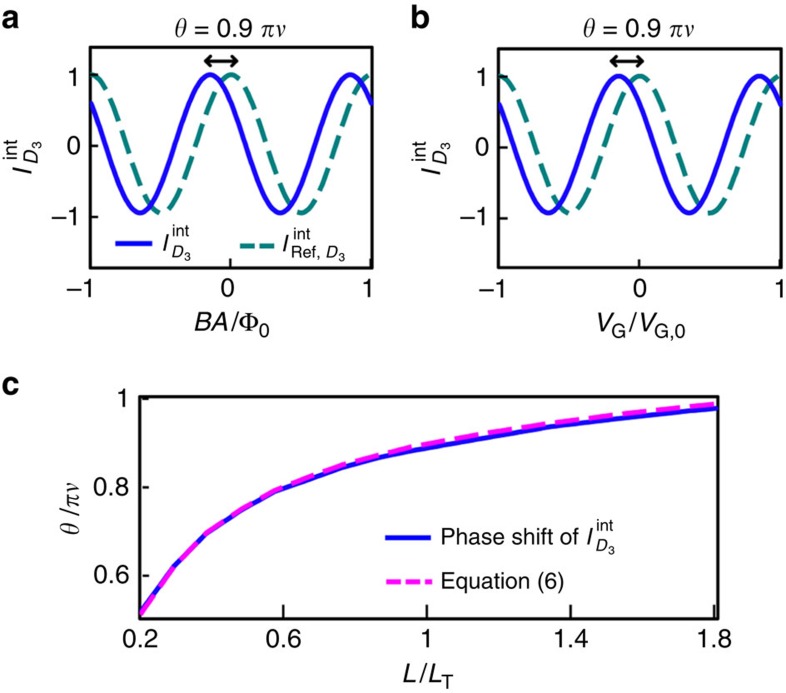
Detection of anyon phase *πν* from interference phase shift *θ*. (**a**) Dependence of 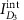 (blue, normalized) and 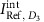
(cyan, normalized) on Φ in the pure Aharonov–Bohm regime and (**b**) their dependence on *V*_G_ in the Coulomb dominated regime. We choose *ν*=1/3, *T*=30 mK, e*V=45 μeV, *L*=3 μm and *v*_p_=10^4^ m s^−1^ (*L*/*L*_*T*_=1.2 and *L*/*L*_*V*_=20); see Supplementary Note 5 for the Coulomb interaction parameter of the regimes. For these parameters, the phase shift *θ* between 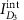 and 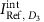 is 0.9*πν*. (**c**) Dependence of *θ* on *T*. The same parameters (except *T*) with (**a**) and (**b**) are chosen. *θ*→*πν* as *T* increases (yet 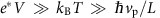). In both the pure Aharonov–Bohm regime and the Coulomb-dominated regime, the same numerical result (blue curve) of *θ*(*T*), which agrees with equation (6) (magenta), is obtained.

For 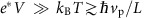, we numerically compute 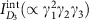 in Fig. 3, combining our theory with the capacitive interaction model^25^ that successfully describes thermally fluctuating bulk anyons and the interaction (see the Method and Supplementary Note 5). We find the gate-voltage dependence of 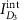
∝cos(2*πV*_G_/*V*_G,0_+*θ*) with periodicity *V*_G,0_ in the Coulomb-dominated regime and 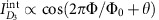 in the pure Aharonov–Bohm limit; here the periodicity of the Φ dependence is Φ_0_≡*h*/*e* rather the period 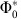 of equation (5), because of the fluctuation of the number of bulk anyons^9^,^19^. In both the regimes, the interference processes discussed before (Fig. 2) appear in the same manner; hence, *θ* satisfies the analytic expression in equation (6) (*cf*. Fig. 3c).

### How to measure the phase *θ*

Experimental measurements of *θ* can be affected by possible side effects, including the external-parameter (magnetic fields, gate voltages and bias voltages) dependence of the size, shape, QPC tunnelling and bulk anyon excitations. Below we propose how to detect *θ* with avoiding the side effects, using the setup in Fig. 2a.

The phase *θ* is experimentally measurable, by comparing 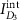 with a reference current 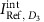. 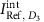
is measured at *D*_3_ in the same setup under the same external parameters (temperature, gate voltages, magnetic field and so on) with 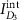, but with applying infinitesimal bias voltage *V*_ref_/2 to *S*_2_ and −*V*_ref_/2 to *S*_3_ and keeping *S*_1_ and all *D*_*i*_'s grounded^9^ (*cf*. Supplementary Note 4). In any regimes 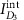 shows the same interference pattern with 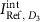, but is phase-shifted from 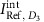
by *θ*; 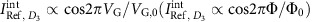 in the Coulomb-dominated (pure Aharonov–Bohm) limit. Importantly, the side effects modify 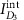 and 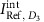
in the same manner; hence, the phase shift between the patterns remains as *θ*.

The fractional statistics phase is directly and unambiguously identifiable in experiments, by observing *θ*→*πν* at 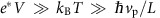 with excluding the side effects as above, or one applies the fit function of arctan [*A*_1_/(1+*A*_2_/*T*)] with fit parameters *A*_1_ and *A*_2_ to measured data of *θ*(*T*) and extracts arctan *A*_1_=*πν* from the fit (*cf*. the second line of equation (6)). Observation of *θ*=*πν* or *θ*(*T*) will suggest a strong evidence of anyon braiding and topological bubbles.

The parameters in Fig. 3 are experimentally accessible^17^,^20^,^21^,^22^,^23^. For the QPCs, there are constraints (i) that the number of voltage-biased anyons injected through QPC1 is at most one in the interferometer loop at any instance (to ensure that the braiding phase of a topological vacuum bubble is 2*πν*), (ii) that the anyon tunnelling probabilities at QPC2 and QPC3 are sufficiently small (to ensure that the double winding of an anyon along the interferometer loop is negligible) and (iii) that anyon tunnelling (rather than electron tunnelling) occurs at the QPCs. The constraint (i) is satisfied when the anyon tunnelling probability at QPC1 is <*ℏv*_p_/(2Le*V), which is ∼0.05 under the parameters. To achieve the constraints (ii) and (iii), each tunnelling probability of QPC2 and QPC3 is typically set to be 0.4 in experiments^22^,^29^; then the amplitude of the double winding is smaller than that of the single winding by the factor 0.4 exp(−2*L*/*L*_*T*_), which is ∼0.04 at 30 mK. With the constraints we estimate the amplitude of 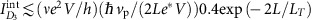, which is 1.5 pA at 30 mK and 0.6 pA at 40 mK under the parameters. It is noteworthy that *θ*=0.9*πν* is reached at 30 mK, while *θ*=0.95*πν* at 40 mK under the parameters. The estimation is within a measurable range in experiments, where current 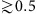 pA is well detectable^30^.

We remark that the above strategy of detecting the fractional statistics phase is equally applicable to the more general quantum Hall regime^22^ of filling factor *ν*′=*ν*+*ν*_0_, in which the edge channels from the integer filling *ν*_0_ are fully transmitted through the QPCs, while the channel from the fractional filling *ν* forms the interferometry in Fig. 2. For this case we compute 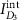 and 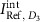, and find that in both of the pure Aharonov–Bohm regime and the Coulomb-dominated regime the interference-pattern phase shift between them is identical to the phase *θ* of the *ν*_0_=0 case discussed in equation (6) and Fig. 3 (*cf*. Supplementary Note 5).

Certain anyonic vacuum bubbles involve topological braiding and affect physical observables surprisingly, contrary to vacuum bubbles of bosons and fermions. They can be detected with current experiment tools, which will provide an unambiguous evidence of anyonic fractional statistics. We expect that they are relevant also for other filling fractions *ν*=*p*/(2*np*+1), non-Abelian anyons^17^,^21^ and topological quantum computation setups^31^.

## Methods

### Hamiltonian for the interferometer

We present the Hamiltonian for the setup. We recall the chiral Luttinger liquid theory for FQH edges.

The Hamiltonian *H*=∑_*i*_*H*_edge,*i*_+*H*_tun_ for the interferometer in Fig. 2a consists of *H*_edge,*i*_ for edge channel *i* and *H*_tun_ for anyon tunnelling at QPCs. Edge channel 1 is biased by *V* and its Hamiltonian, employing the bosonization^26^ for chiral Luttinger liquids, is given by


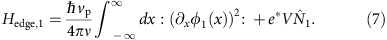


For the other unbiased channels, 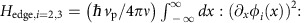 Here, e*>0, 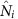 is the anyon number operator of channel *i* and *φ*_*i*_(*x*) is the bosonic field of channel *i* at position *x*, which describes the plasmonic excitation of anyons. The tunnelling Hamiltonian is 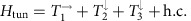, 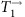 is the operator from Edge channel 1 to 2 at QPC 1, 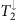 from Edge 2 to 3 at QPC2 and 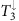 from Edge 2 to 3 at QPC3. These are written as


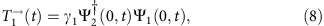



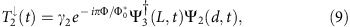



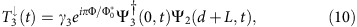


where 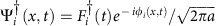 creates an (particle-like) anyon at position *x* and time *t* on Edge *i*, *a* is the short-length cutoff, *γ*_*i*_ is the tunnelling strength at QPC *i* (chosen as real) and the Aharonov–Bohm flux Φ enclosed by Edges *i*=2,3, QPC2 and QPC3 is attached to 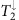 and 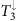, respectively, under certain gauge transformation; the dynamical phase common to the all edge channels is absorbed to 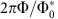. The Klein factor 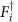 increases the number of anyons on Edge *i* by 1 and satisfies 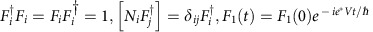 and 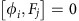.

The exchange rule in equation (1) is described by *φ*_*i*_ and *F*_*i*_. On Edge *i*, it is satisfied by


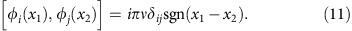


The exchange rule between anyons on different edges is achieved with the commutators of *F*_*i*_,


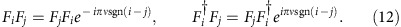


**Figure 4:**
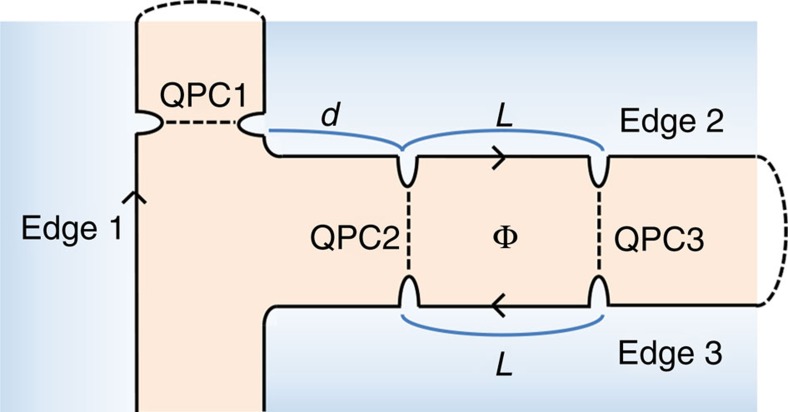
Extended edge channel scheme. It is obtained by connecting the edge channels of the setup in Fig. 2a. The connection is represented by dashed arcs, whereas anyon propagation direction and anyon tunnelling at QPCs are represented by arrows and dashed lines, respectively.

A conventional way^32^ for obtaining the commutators is to think of an extended edge connecting the different channel segments with no twist (*cf*. Fig. 4). The connection should preserve the chiral propagation direction of the channels. The exchange rule 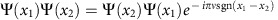 of anyons of the extended edge agrees with equations (11) and (12).

We consider the regime of weak tunnelling of anyons and treat *H*_tun_ as a perturbation on ∑_*i*_*H*_edge,*i*_. Perturbation theory is applicable^24^ in the renormalization group sense, when e*V and *k*_B_*T* are higher than *Cγ*^1/(1−*ν*)^, *C* being a non-universal constant.

The current 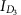 is expressed as 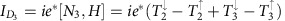. 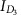 is decomposed, 
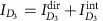
, into direct current 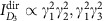 and interference current 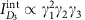 depending on Φ (the leading-order contribution). Equations (3) and (4) are obtained by employing Keldysh Green's function technique with semiclassical approximation (see Supplementary Fig. 1 and Supplementary Notes 1, 2 and 3).

### Coulomb interaction

In the presence of Coulomb interaction between bulk anyons and edge channels, we compute 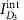, combining our chiral Luttinger liquid theory with the capacitive interaction model^25^ that successfully describes the Coulomb-dominated regime. The interferometer Hamiltonian *H*=∑_*i*_*H*_edge,*i*_+*H*_tun_ is modified by the Coulomb interaction as


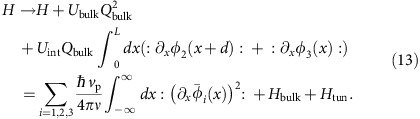


Here, 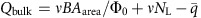 is the number of the net charges localized within the interferometer bulk (inside the interference loop), *A*_area_ is the area of the interferometer, *N*_L_ is the net number of quasiparticles minus quasiholes and 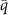 is the number of positive background charges induced by the gate voltage applied to the interferometer. *U*_int_ is the strength of Coulomb interaction between the charges of the interferometer edge and the charges localized in the interferometer bulk, and *U*_bulk_ is the strength of interaction between the bulk charges. In the second equality of equation (13), we introduce a boson field 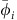 for each Edge *i*, 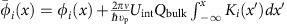, where *K*_2_(*x*)=1 for *d*<*x*<*d*+*L*, *K*_3_(*x*)=1 for 0<*x*<*L* and *K*_*i*_(*x*)=0 otherwise. The second term of 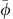 describes the charges 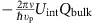 induced per unit length by the interaction. In equation (13), the Hamiltonian is quadratic in 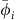 and has 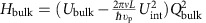. It is noteworthy that 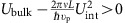. The main interference signal 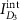 and the reference signal 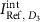
are computed by taking ensemble average over the thermal fluctuations of *N*_L_ (see Supplementary Note 5).

## Additional information

**How to cite this article:** Han, C. *et al.* Topological vacuum bubbles by anyon braiding. *Nat. Commun.* 7:11131 doi: 10.1038/ncomms11131 (2016).

## Supplementary Material

Supplementary Information Supplementary Figures 1-2, Supplementary Notes 1-6, Supplementary References
