# Supplementary material for: Topological vacuum bubbles by anyon braiding
Source: Nat Commun. 2016 Mar 31;7:11131. doi: 10.1038/ncomms11131 (PMC4821888; doi:10.1038/ncomms11131)
Supplement: Supplementary Information — Supplementary Figures 1-2, Supplementary Notes 1-6, Supplementary References [file ncomms11131-s1.pdf]

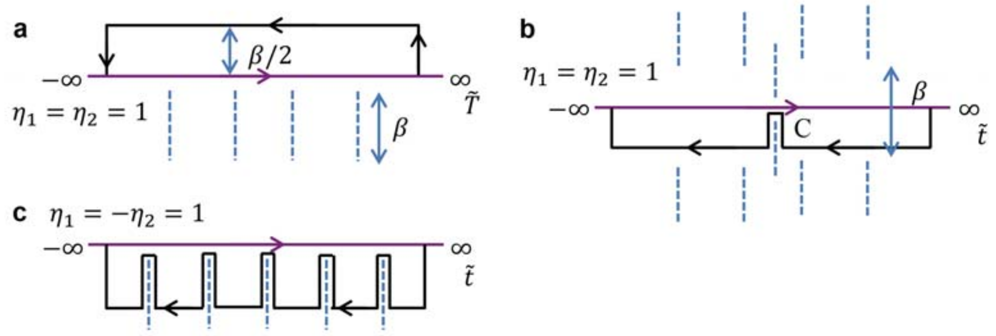

Supplementary Figure 1. Integration contour for the terms of $\eta_1 = \eta_2 = 1$ and $\eta_1 = -\eta_2 = 1$ in Supplementary Equation 3. Magenta lines represent the real-axis integral line, while the black line a proper contour for contour integral in the complex plane of t. Dashed blue lines show branch-cut lines.

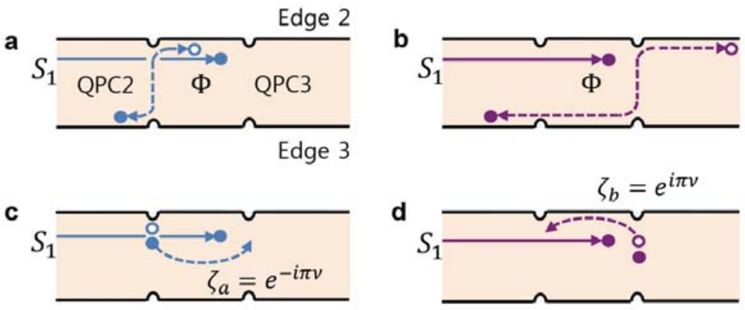

Supplementary Figure 2. Braiding phase of Type II process. (**a**) and (**b**), The two interfering paths of the Type II process in Fig. 2. (**c**) and (**d**), Paths equivalent, respectively, to those in (**a**) and (**b**) in the gauge chosen in parallel to the extended edge scheme in Fig. 4 of the main text. In the interference of the two paths (**a**) and (**b**), a thermal anyon (whose trajectory is represented by dashed lines) excited at QPC2 or QPC3 *effectively* winds around a voltage-biased anyon (solid line) injected from Edge 1 via QPC1. The effective winding results in the braiding phase factor $\zeta_a^* \zeta_b = e^{i2\pi\nu}$. The phase factors ζ_a and ζ_b are derived from the commutation rules in Eqs. (11) and (12). For the details, see the Supplementary Note 6.

Supplementary Note 1 : Green's function. We provide the Green's function of bosonic fields ϕ_i. It is defined as

$$G(t,x) = \langle \phi_i(t,x)\phi_i(0,0)\rangle - \langle \phi_i(0,0)\phi_i(0,0)\rangle, \tag{1}$$

where $\langle \cdots \rangle$ is the ensemble average under the bare Hamiltonian $H_{\text{edge},i}$. The expression of G is known [1] as $G_\beta(t,x) = -\nu \ln \left[\frac{\sin(\pi(\tau_0 + i(t-x))/\beta)}{\pi\tau_0/\beta}\right]$, where $\hbar/\tau_0$ is the infrared energy cutoff and we use $v_{\rm p} \equiv 1$ and $\beta = (k_B T)^{-1}$. At zero temperature, $G(x,t) = -\nu \ln(\tau_0 + i(t-x)/\tau_0)$.

To describe the non-equilibrium situation by the voltage V at edge 1, we use the Green's function on the standard Keldysh contour with Keldysh ordering T_K. The contour has two branches, the upper one denoted by Keldysh index $\eta = 1$ and the lower of $\eta = -1$. For two operators A and B on the upper branch, $T_K(AB) = AB$ if B is the earlier event than A, and $T_K(AB) = BA$ otherwise; it is correct not to attach the phase factor for the exchange statistics in the definition of Keldysh ordering [2]. For A and B on the lower, $T_K(AB) = BA$ if B is earlier than A, and $T_K(AB) = AB$ otherwise. For A on the upper and B on the lower, $T_K(AB) = BA$. For A on the lower and B on the upper, $T_K(AB) = AB$. The Keldysh Green's function $G_{\eta\eta_0}(t,x) \equiv \langle T_K[\phi_i(t^\eta, x)\phi_i(0^{\eta_0}, 0)]\rangle - \langle \phi_i(0,0)\phi_i(0,0)\rangle$ is obtained as [2]

$$G_{\eta\eta_0}(t,x) = -\nu \ln \frac{\sin\left(\pi(\tau_0 + i\chi_{\eta\eta_0}(t)(t-x))/\beta\right)}{\pi\tau_0/\beta}, \tag{2}$$

where $\chi_{\eta_1\eta_2}(t_1 - t_2) = [(\eta_1 + \eta_2)/2]\text{sgn}(t_1 - t_2) - (\eta_1 - \eta_2)/2$ and η_0 and η are Keldysh indexes.

Supplementary Note 2 : Perturbation theory. We compute the charge current I_{D_3} at drain D_3 by the voltage V at edge 1 up to order $\gamma_1^2\gamma_2\gamma_3$, employing perturbation theory and the Keldysh Green function in Supplementary Equation 2. Hereafter, we occasionally use $\omega_0 \equiv e^* V$, $\beta \equiv (k_\mathrm{B}T)^{-1}$, $v_\mathrm{p} \equiv 1$, and $\hbar \equiv 1$.

The current I_{D_3} is expressed as $I_{D_3} = ie^*[N_3, H] = ie^*(\langle T_2^{\downarrow}\rangle - \langle T_2^{\uparrow}\rangle + \langle T_3^{\downarrow}\rangle - \langle T_3^{\uparrow}\rangle)$. I_{D_3} is decomposed, $I_{\mathrm{D}_3} = I_{\mathrm{D}_3}^{\mathrm{dir}} + I_{\mathrm{D}_3}^{\mathrm{int}}$, into direct current $I_{\mathrm{D}_3}^{\mathrm{dir}} \propto \gamma_1^2\gamma_2^2$, $\gamma_1^2\gamma_3^2$, and interference current $I_{\mathrm{D}_3}^{\mathrm{int}} \propto \gamma_1^2\gamma_2\gamma_3$ depending on Φ (the leading-order contribution).

We focus on the contribution of $\langle T_2^{\downarrow}\rangle$ onto $I_{\mathrm{D}_3}^{\mathrm{int}} \propto \gamma_1^2\gamma_2\gamma_3$, which is denoted by $\langle T_2^{\downarrow,\mathrm{int}}\rangle$. It is

$$\langle T_2^{\downarrow,\mathrm{int}}\rangle = \sum_{\eta,\eta_0\eta_1\eta_2=\pm1} \eta\eta_1\eta_2 \int_{-\infty}^{\infty} dt_1 \int_{-\infty}^{\infty} dt_2 \int_{-\infty}^{\infty} dt \langle T_K[T_2^{\downarrow}(0^{\eta_0}) T_1^{\rightarrow}(t_1^{\eta_1}) T_1^{\leftarrow}(t_2^{\eta_2}) T_3^{\uparrow}(t^{\eta})]\rangle.$$

$\langle T_K[T_2^{\downarrow}(0^{\eta_0}) T_1^{\rightarrow}(t_1^{\eta_1}) T_1^{\leftarrow}(t_2^{\eta_2}) T_3^{\uparrow}(t^{\eta})]\rangle/\gamma_1^2\gamma_2\gamma_3$ is decomposed into two parts,

$$\begin{aligned}
&\langle T_K[\Psi_3^\dagger(0^{\eta_0}, L)\Psi_2(0^{\eta_0}, d)\Psi_2^\dagger(t_1^{\eta_1}, 0)\Psi_1(t_1^{\eta_1}, 0)\Psi_1^\dagger(t_2^{\eta_2}, 0)\Psi_2(t_2^{\eta_2}, 0)\Psi_2^\dagger(t^{\eta}, d+L)\Psi_3(t^{\eta}, 0)]\rangle \\
=&\langle T_K[e^{-i\phi_3(0^{\eta_0},L)} e^{i\phi_2(0^{\eta_0},d)} e^{-i\phi_2(t_1^{\eta_1},0)} e^{i\phi_1(t_1^{\eta_1},0)} e^{-i\phi_1(t_2^{\eta_2},0)} e^{i\phi_2(t_2^{\eta_2},0)} e^{-i\phi_2(t^{\eta},d+L)} e^{i\phi_3(t^{\eta},0)}]\rangle \\
&\times \langle T_K[F_3^\dagger(0^{\eta_0})F_2(0^{\eta_0})F_2^\dagger(t_1^{\eta_1})F_1(t_1^{\eta_1})F_1^\dagger(t_2^{\eta_2})F_2(t_2^{\eta_2})F_2^\dagger(t^{\eta})F_3(t^{\eta})]\rangle.
\end{aligned}$$

We compute the former part, using the identity (valid when the Hamiltonian of B's is quadratic) for bosonic operators B's, $\langle T_K[e^{B_1(t_1)}e^{B_2(t_2)}...e^{B_n(t_n)}]\rangle = e^{\frac{1}{2}\sum_{j=1}^n \langle T_K[B_j(t_j)^2]\rangle + \sum_{i<j}\langle T_K[B_i(t_i)B_j(t_j)]\rangle}$,

$$\begin{aligned}
&\langle T_K[e^{-i\phi_3(0^{\eta_0},L)} e^{i\phi_2(0^{\eta_0},d)} e^{-i\phi_2(t_1^{\eta_1},0)} e^{i\phi_1(t_1^{\eta_1},0)} e^{-i\phi_1(t_2^{\eta_2},0)} e^{i\phi_2(t_2^{\eta_2},0)} e^{-i\phi_2(t^{\eta},d+L)} e^{i\phi_3(t^{\eta},0)}]\rangle \\
&= e^{2G_{\eta_1\eta_2}(t_1-t_2,0)+G_{\eta_1\eta_0}(t_1,-d)-G_{\eta_1\eta}(t_1-t,-d-L)+G_{\eta_2\eta}(t_2-t,-d-L)-G_{\eta_2\eta_0}(t_2,-d)+G_{\eta\eta_0}(t,-L)+G_{\eta\eta_0}(t,L)},
\end{aligned}$$

in which the Keldysh Green's functions $G_{\eta\eta_0}$ in Supplementary Equation 2 appear. The latter Klein-factor part is

$$\begin{aligned}
&\langle T_K[F_3^\dagger(0^{\eta_0})F_2(0^{\eta_0})F_2^\dagger(t_1^{\eta_1})F_1(t_1^{\eta_1})F_1^\dagger(t_2^{\eta_2})F_2(t_2^{\eta_2})F_2^\dagger(t^{\eta})F_3(t^{\eta})]\rangle \\
&= e^{-i\omega_0(t_1-t_2)} \frac{\exp[i\pi\nu\{\chi_{\eta_1\eta_0}(t_1)+\chi_{\eta_2\eta}(t_2-t)\}/2]}{\exp[i\pi\nu\{\chi_{\eta_2\eta_0}(t_2)+\chi_{\eta_1\eta}(t_1-t)\}/2]}.
\end{aligned}$$

It is obtained, by using $F_1(t) = F_1(0)e^{-ie^*Vt/\hbar}$, $F_2(t) = F_2(0)$, $F_3(t) = F_3(0)$, $F_i^\dagger F_i = F_i F_i^\dagger = 1$, and the exchange statistics rule in Eq. (12). The final expression is

$$\begin{aligned}
\langle T_2^{\downarrow,\mathrm{int}}\rangle =& \gamma_1^2\gamma_2\gamma_3 \sum_{\eta_1,\eta_2,\eta=\pm1} \int_{-\infty}^{0} dt \int_{-\infty}^{\infty} dt_2 \int_{-\infty}^{\infty} dt_1 \frac{\eta_1\eta_2\eta e^{-i2\pi\Phi/\Phi_0^*} e^{-i\omega_0(t_1-t_2)}}{\left(\frac{\beta}{\pi}\sin(\frac{\pi}{\beta}(\tau_0 + i\chi_{\eta_1\eta_2}(t_1-t_2)(t_1-t_2)))\right)^{2\nu}} \\
&\times \frac{1}{\left(\frac{\beta}{\pi}\sin(\frac{\pi}{\beta}(\tau_0 - i\eta(t+L)))\right)^{\nu}\left(\frac{\beta}{\pi}\sin(\frac{\pi}{\beta}(\tau_0 - i\eta(t-L)))\right)^{\nu}} \\
&\times \frac{\left(\sin(\frac{\pi}{\beta}(\tau_0 - i\eta_1(t_1-t+d+L)))\right)^{\nu}\left(\sin(\frac{\pi}{\beta}(\tau_0 - i\eta_2(t_2+d)))\right)^{\nu}}{\left(\sin(\frac{\pi}{\beta}(\tau_0 - i\eta_1(t_1+d)))\right)^{\nu}\left(\sin(\frac{\pi}{\beta}(\tau_0 - i\eta_2(t_2-t+d+L)))\right)^{\nu}}.
\end{aligned}$$

Computing the other terms in the same way, we obtain

$$\begin{aligned}
I_{D_3}^{\mathrm{int}} =& \alpha\gamma_1^2\gamma_2\gamma_3 \sum_{\epsilon\eta_1,\eta_2,\eta=\pm1} \int_{-\infty}^{\infty} dt \int_{-\infty}^{\infty} dt_2 \int_{-\infty}^{\infty} dt_1 \frac{\epsilon\eta_1\eta_2\eta e^{-i\epsilon 2\pi\Phi/\Phi_0^*} e^{-i\omega_0(t_1-t_2)}}{\left(\frac{\beta}{\pi}\sin(\frac{\pi}{\beta}(\tau_0 + i\chi_{\eta_1\eta_2}(t_1-t_2)(t_1-t_2)))\right)^{2\nu}} \\
&\times \frac{1}{\left(\frac{\beta}{\pi}\sin(\frac{\pi}{\beta}(\tau_0 - i\eta(t+L)))\right)^{\nu}\left(\frac{\beta}{\pi}\sin(\frac{\pi}{\beta}(\tau_0 - i\eta(t-L)))\right)^{\nu}} \\
&\times \left[\frac{\left(\sin(\frac{\pi}{\beta}(\tau_0 - i\eta_1(t_1-t+d+L)))\right)^{\nu}\left(\sin(\frac{\pi}{\beta}(\tau_0 - i\eta_2(t_2+d)))\right)^{\nu}}{\left(\sin(\frac{\pi}{\beta}(\tau_0 - i\eta_1(t_1+d)))\right)^{\nu}\left(\sin(\frac{\pi}{\beta}(\tau_0 - i\eta_2(t_2-t+d+L)))\right)^{\nu}}\right]^{\epsilon},
\end{aligned} \tag{3}$$

where $\alpha \equiv (e^*/\hbar)a^{4\nu}/[(\hbar v_\mathrm{p})^{4\nu}(2\pi a)^4]$. Supplementary Equation 3 is written as $I_{\mathrm{D}_3}^{\mathrm{int}} \propto \cos(2\pi\frac{\Phi}{\Phi_0^*}+\theta)$ in Eq. (5),

$$\theta = \arg[\sum_{\eta_1,\eta_2,\eta=\pm1}\int_{-\infty}^{\infty}dt\int_{-\infty}^{\infty}dt_2\int_{-\infty}^{\infty}dt_1\frac{\eta_1\eta_2\eta e^{-i\omega_0(t_1-t_2)}}{\left(\frac{\beta}{\pi}\sin(\frac{\pi}{\beta}(\tau_0+i\chi_{\eta_1\eta_2}(t_1-t_2)(t_1-t_2)))\right)^{2\nu}}\frac{1}{\left(\frac{\beta}{\pi}\sin(\frac{\pi}{\beta}(\tau_0-i\eta(t+L)))\right)^{\nu}}$$

$$\times\frac{1}{\left(\frac{\beta}{\pi}\sin(\frac{\pi}{\beta}(\tau_0-i\eta(t-L)))\right)^{\nu}}\frac{\left(\sin(\frac{\pi}{\beta}(\tau_0-i\eta_1(t_1-t+d+L)))\right)^{\nu}\left(\sin(\frac{\pi}{\beta}(\tau_0-i\eta_2(t_2+d)))\right)^{\nu}}{\left(\sin(\frac{\pi}{\beta}(\tau_0-i\eta_1(t_1+d)))\right)^{\nu}\left(\sin(\frac{\pi}{\beta}(\tau_0-i\eta_2(t_2-t+d+L))\right)^{\nu}}]. \quad (4)$$

We omit the expression of the direct-current part $I_{\mathrm{D}_3}^{\mathrm{dir}}$ of I_{D_3} (of the order of $\gamma_1^2\gamma_2^2$ and $\gamma_1^2\gamma_3^2$). The whole expression of I_{D_3} (up to the order of $\gamma_1^2\gamma_2\gamma_3$) is zero at $V=0$, as expected.

Supplementary Note 3 : Semiclassical Approximation. We derive Eqs. (3), (4) and (6) in the main text, by performing the integrals in Supplementary Equation 3 (or those in Supplementary Equation 4) in the semiclassical regime of $e^*V \gg k_\mathrm{B}T \gg \hbar v_\mathrm{p}/L$.

In the semiclassical regime, the wave packet size of an injected anyon from S_1 is $W \sim \hbar v_\mathrm{p}/(e^*V) \ll L$, hence, the anyon is thought of as particle-like (rather than wave-like). And the injection of hole-like anyons (induced by thermal fluctuations) from S_1 can be ignored. Thermally excited virtual anyons also have size much smaller than L. Then, the braiding of the anyons results in the well-defined exchange statistics phase of $\pi\nu$.

In Supplementary Equation 3, there are 16 configurations of η_1, η_2, η, and ϵ. η_1 and η_2 $(=\pm1)$ are Keldysh indexes for tunneling at QPC1, while η is for tunneling at QPC2 or QPC3. $\epsilon=\pm1$ represents the winding direction of anyons around Aharonov-Bohm flux Φ. The terms of Supplementary Equation 3 are classified, depending on whether $\eta_1=\eta_2$. In the regime of $e^*V \gg k_\mathrm{B}T \gg \hbar v_\mathrm{p}/L$, some terms of $\eta_1\neq\eta_2$ are represented by connected Feynman diagrams, and contribute to Type I-1 and Type I-2. The other terms of $\eta_1\neq\eta_2$ and all the terms of $\eta_1=\eta_2$ are represented by vacuum bubbles.

Some of the vacuum bubbles of $\eta_1\neq\eta_2$ describe the virtual anyonic excitations winding around a real anyon injected from S_1 (similarly to Fig. 1a in the main text). Each of such bubble diagrams has a partner diagram among the terms of $\eta_1=\eta_2$, in which a vacuum bubble does not wind around the real anyon (similarly to Fig. 1b in the main text); each of the diagrams of $\eta_1=\eta_2$ is decomposed into two disconnected and independent subdiagrams, one describing the real anyon, and the other for the virtual anyonic excitations not winding around the real anyon. A bubble diagram of $\eta_1\neq\eta_2$ winding around a real anyon, and its partner diagram of $\eta_1=\eta_2$ cancel each other. The cancellation is only partial, resulting in the interference current of Type II and Type III in Eqs. (3) and (4), and disobeying the linked cluster theorem; the cancellation is exact in the case of bosons or fermions. Note that there are the terms of $\eta_1\neq\eta_2$ not involving any braiding effect between real anyons and virtual anyons. These terms show divergence, but are fully canceled by their partner diagrams of $\eta_1=\eta_2$, satisfying the linked cluster theorem.

We now further compute Supplementary Equation 3 in the limit of $e^*V \gg k_\mathrm{B}T \gg \hbar v_\mathrm{p}/L$. For example,

$$\text{the term of }(\eta_1=\eta_2=\pm1,\epsilon=1)\text{ in Supplementary Equation 3} = 2\alpha\gamma_1^2\gamma_2\gamma_3\sum_{\eta,\eta_1}\eta\int_{-\infty}^{\infty}dt\int_{-\infty}^{\infty}d\tilde{t}\int_{-\infty}^{\infty}d\tilde{T}$$

$$\times\frac{e^{-i2\pi\Phi/\Phi_0^*}e^{-i\omega_0 2\tilde{t}}}{\left(\frac{\beta}{\pi}\sin(\frac{\pi}{\beta}(\tau_0+i\eta_1\mathrm{sgn}(\tilde{t})2\tilde{t}))\right)^{2\nu}\left(\frac{\beta}{\pi}\sin(\frac{\pi}{\beta}(\tau_0-i\eta(t+L)))\right)^{\nu}\left(\frac{\beta}{\pi}\sin(\frac{\pi}{\beta}(\tau_0-i\eta(t-L)))\right)^{\nu}}$$

$$\times\left[\frac{\left(\sin(\frac{\pi}{\beta}(\tau_0-i\eta_1(\tilde{T}+\tilde{t}-t+d+L)))\right)^{\nu}\left(\sin(\frac{\pi}{\beta}(\tau_0-i\eta_1(\tilde{T}-\tilde{t}+d)))\right)^{\nu}}{\left(\sin(\frac{\pi}{\beta}(\tau_0-i\eta_1(\tilde{T}+\tilde{t}+d)))\right)^{\nu}\left(\sin(\frac{\pi}{\beta}(\tau_0-i\eta_1(\tilde{T}-\tilde{t}-t+d+L))\right)^{\nu}}\right]$$

$$\simeq 2\alpha\gamma_1^2\gamma_2\gamma_3\sum_{\eta,\eta_1}\eta\int_{-\infty}^{\infty}dt\int_{-\infty}^{\infty}d\tilde{T}\int_{C}d\tilde{t}$$

$$\times\frac{e^{-i2\pi\Phi/\Phi_0^*}e^{-i\omega_0 2\tilde{t}}\left[1+\frac{2\pi\nu}{\beta}\tilde{t}\,\mathrm{sgn}(t-L)\left\{\mathrm{sgn}(\tilde{T}-t+d+L)\mathrm{sgn}(\tilde{T}+d)-1\right\}\right]}{\left(\frac{\beta}{\pi}\sin(\frac{\pi}{\beta}(\tau_0+i\eta_1\mathrm{sgn}(\tilde{t})2\tilde{t}))\right)^{2\nu}\left(\frac{\beta}{\pi}\sin(\frac{\pi}{\beta}(\tau_0-i\eta(t+L)))\right)^{\nu}\left(\frac{\beta}{\pi}\sin(\frac{\pi}{\beta}(\tau_0-i\eta(t-L)))\right)^{\nu}}$$

In the first equality, we put $\tilde{t}=(t_1-t_2)/2$ and $\tilde{T}=(t_1+t_2)/2$ into Supplementary Equation 3. In the second, we change the integral range of $\tilde{T}$ from $\tilde{T}\in(-\infty,\infty)$ to $\tilde{T}\in(-\infty+i\eta_1\beta/2,\infty+i\eta_1\beta/2)$ using the contour shown in

Supplementary Figure 1a, and that of $\tilde{t}$ from $\tilde{t} \in (-\infty, \infty)$ to the vertical parts C near $\mathrm{Re}(\tilde{t}) = 0$ of the contour in Supplementary Figure 1b. Note that the integral of $\tilde{t}$ along the horizontal black line of Supplementary Figure 1b is negligible in the limit of $e^* V \gg k_\mathrm{B} T \gg \hbar v_\mathrm{p}/L$. Then

$$\begin{aligned}
&\text{Sum of the } (\eta_1 = \eta_2 = 1, \epsilon = 1) \text{ term and the } (\eta_1 = \eta_2 = -1, \epsilon = 1) \text{ term} \\
&\simeq \alpha \gamma_1^2 \gamma_2 \gamma_3 \int_{-\infty}^{\infty} dt \int_{-\infty}^{\infty} d\tilde{T} \left[1 + \frac{2\pi i \nu (1-2\nu)\ \mathrm{sgn}(t-L)}{\omega \beta} \left\{ \mathrm{sgn}(\tilde{T} - t + d + L) \mathrm{sgn}(\tilde{T} + d) - 1 \right\} \right] \\
&\qquad \times \sum_\eta \eta \frac{A_1 e^{-i 2\pi \Phi / \Phi_0^*}}{\left(\frac{\beta}{\pi} \sin(\frac{\pi}{\beta}(\tau_0 - i\eta(t+L))) \right)^\nu \left(\frac{\beta}{\pi} \sin(\frac{\pi}{\beta}(\tau_0 - i\eta(t-L))) \right)^\nu},
\end{aligned} \tag{5}$$

where $A_1 = \int_{-\infty}^{\infty} d\tilde{t} e^{-i\omega_0 \tilde{t}} \left(\frac{\beta}{\pi} \sin(\frac{\pi}{\beta}(\tau_0 - i\tilde{t})) \right)^{-2\nu} \simeq \frac{2\pi}{\Gamma(2\nu)} \omega_0^{2\nu - 1}$ and $\Gamma(2\nu)$ is the Gamma function. The divergence of Supplementary Equation 5 is fully canceled by some terms of $\eta_1 \neq \eta_2$. Next, we consider

$$\begin{aligned}
&\text{the term of } (\eta_1 = 1, \eta_2 = -1, \epsilon = 1) = 2\alpha \gamma_1^2 \gamma_2 \gamma_3 \sum_\eta (-\eta) \int_{-\infty}^{\infty} dt \int_{-\infty}^{\infty} d\tilde{T} \int_{-\infty}^{\infty} d\tilde{t} \\
&\times \frac{e^{-i2\pi\Phi/\Phi_0^*} e^{-i\omega_0 2\tilde{t}}}{\left(\frac{\beta}{\pi} \sin(\frac{\pi}{\beta}(\tau_0 - i2\tilde{t})) \right)^{2\nu} \left(\frac{\beta}{\pi} \sin(\frac{\pi}{\beta}(\tau_0 - i\eta(t+L))) \right)^\nu \left(\frac{\beta}{\pi} \sin(\frac{\pi}{\beta}(\tau_0 - i\eta(t-L))) \right)^\nu} \\
&\times \left[\frac{\left(\sin(\frac{\pi}{\beta}(\tau_0 - i(\tilde{T} + \tilde{t} - t + d + L))) \right)^\nu \left(\sin(\frac{\pi}{\beta}(\tau_0 + i(\tilde{T} - \tilde{t} + d))) \right)^\nu}{\left(\sin(\frac{\pi}{\beta}(\tau_0 - i(\tilde{T} + \tilde{t} + d))) \right)^\nu \left(\sin(\frac{\pi}{\beta}(\tau_0 + i(\tilde{T} - \tilde{t} - t + d + L))) \right)^\nu} \right] \\
&\simeq \alpha \gamma_1^2 \gamma_2 \gamma_3 \int_{-\infty}^{\infty} dt \left(-A_2^2 e^{i\pi\nu \mathrm{sgn}(t-L)} - A_2^2 e^{i\omega_0 (t-L)} - A_1 \int_{-\infty}^{\infty} d\tilde{T} e^{-i\pi\nu(\mathrm{sgn}(\tilde{T} - t + d + L) - \mathrm{sgn}(\tilde{T} + d))} \right. \\
&\left. \times \left[1 + \frac{2\pi i \nu (1 - 2\nu)\ \mathrm{sgn}(t-L)}{\omega\beta} \left\{ \mathrm{sgn}(\tilde{T} - t + d + L) \mathrm{sgn}(\tilde{T} + d) - 1 \right\} \right] \right) \\
&\times \sum_\eta \eta \frac{e^{-i2\pi\Phi/\Phi_0^*}}{\left(\frac{\beta}{\pi} \sin(\frac{\pi}{\beta}(\tau_0 - i\eta(t+L))) \right)^\nu \left(\frac{\beta}{\pi} \sin(\frac{\pi}{\beta}(\tau_0 - i\eta(t-L))) \right)^\nu}.
\end{aligned} \tag{6}$$

Here $A_2 = \int_{-\infty}^{\infty} dt e^{-i\omega_0 t} \left(\frac{\beta}{\pi} \sin(\frac{\pi}{\beta}(\tau - it)) \right)^{-\nu} \simeq \frac{2\pi}{\Gamma(\nu)} \omega_0^{\nu-1}$, $\tilde{t} = (t_1 - t_2)/2$, and $\tilde{T} = (t_1 + t_2)/2$. The integral over $\tilde{t}$ is done in the regime of $e^* V \gg k_\mathrm{B} T$, using the contour in Supplementary Figure 1c.

The terms of $(\eta_1 = 1, \eta_2 = -1, \epsilon = 1)$ with coefficient A_2 in Supplementary Equation 6 describe Types I-1 and I-2, which have no counterpart in the terms of $\eta_1 = \eta_2$. The divergence of some terms with coefficient A_1 in Supplementary Equation 6 is fully canceled by the corresponding terms of $\eta_1 = \eta_2$. The remaining terms with A_1 are partially canceled by the corresponding terms of $\eta_1 = \eta_2$, describing Types II and III. Indeed, the difference of the terms with A_1 between Supplementary Equations 5 and 6 is proportional to

$$\begin{aligned}
&- A_1 \int_{-\infty}^{\infty} d\tilde{T} \left(e^{-i\pi\nu(\mathrm{sgn}(\tilde{T} - t + d + L) - \mathrm{sgn}(\tilde{T} + d))} - 1 \right) \\
&\quad \times \left[1 + \frac{2\pi i \nu (1 - 2\nu)\ \mathrm{sgn}(t-L)}{\omega\beta} \left\{ \mathrm{sgn}(\tilde{T} - t + d + L) \mathrm{sgn}(\tilde{T} + d) - 1 \right\} \right] \\
&\quad = -2iA_1 (t - L) e^{i\pi\nu \mathrm{sgn}(t-L)} \left[1 + \frac{2\pi i \nu (1 - 2\nu)\ \mathrm{sgn}(t-L)}{\omega\beta} \right] \sin \pi\nu.
\end{aligned}$$

The difference is proportional to $\sin \pi\nu$, and describes the topological vacuum bubbles corresponding to the braiding discussed in Fig. 1 and Eqs. (3) and (4). The term of $\eta_1 = -1$, $\eta_2 = 1$, and $\epsilon = 1$ describes the injection of hole-like anyons from edge 1 to edge 2, and is negligible in our regime of $e^* V \gg k_\mathrm{B} T$. The terms with $\epsilon = -1$ are obtained in the same way, as they are for the processes of winding Φ in the opposite direction to those of $\epsilon = 1$.

Now we collect all the terms of $I^{\rm int}_{D_3}$,

$$I^{\rm int}_{D_3} = \alpha\gamma_1^2\gamma_2\gamma_3 \sum_\eta (-\eta) \int_{-\infty}^{\infty} dt \frac{1}{\left(\frac{\beta}{\pi}\sin(\frac{\beta}{\pi}(\tau_0 - i\eta(t+L)))\right)^\nu \left(\frac{\beta}{\pi}\sin(\frac{\pi}{\beta}(\tau_0 - i\eta(t-L)))\right)^\nu}$$
$$\times[(-2i\sin(\pi\nu)A_1\{1+f(t)\}e^{i\pi\nu \mathrm{sgn}(t-L)}(t-L) - A_2^2 e^{i\pi\nu\mathrm{sgn}(t-L)} - A_2^2 e^{i\omega_0(t-L)})e^{-i2\pi\Phi/\Phi_0^*}$$
$$- (2i\sin(\pi\nu)A_1\{1-f(t)\}e^{-i\pi\nu\mathrm{sgn}(t-L)}(t-L) - A_2^2 e^{-i\pi\nu\mathrm{sgn}(t-L)} - A_2^2 e^{-i\omega_0(t-L)})e^{i2\pi\Phi/\Phi_0^*}], \qquad (7)$$

where $f(t) = \frac{2\pi i\nu(1-2\nu)\,\mathrm{sgn}(t-L)}{\omega\beta}$. The integral of t is further computed for $k_{\rm B}T \gg \hbar v_{\rm p}/L$; we use $\frac{\beta}{\pi}\sin(\frac{\pi}{\beta}(\tau_0 - it)) \simeq -i\mathrm{sgn}(t)\frac{\beta}{2\pi}e^{\frac{\pi}{\beta}|t|}$ for $t \gg \beta$, $\int_{-\infty}^0 dt e^{\pi\nu t/\beta}\left(\frac{\beta}{\pi}\sin(\frac{\beta}{\pi}(\tau_0+it))\right)^{-\nu} = (\frac{2\pi}{\beta})^\nu \frac{\beta}{2\pi}e^{i\pi\nu/2}\pi\csc(\pi\nu)$, and $\int_{-\infty}^0 dt e^{\pi\nu t/\beta} t \left(\frac{\beta}{\pi}\sin(\frac{\beta}{\pi}(\tau_0+it))\right)^{-\nu} = (\frac{2\pi}{\beta})^\nu(\frac{\beta}{2\pi})^2\pi e^{i\pi\nu/2}(\psi(1)-\psi(\nu))\csc(\pi\nu)$, where $\psi(x)$ is the polygamma function. The integration leads to Eq. (3),

$$\begin{aligned}
I^{\text{I-1}}_{\rm D_3} &= \frac{16\pi^3(2\pi)^\nu\alpha}{\Gamma(\nu)^3}\gamma_1^2\gamma_2\gamma_3(e^*V)^{3\nu-3}(k_{\rm B}T)^\nu\cos(e^*VL/\hbar v_{\rm p} - \pi\nu/2) \\
&\quad\times e^{-2L/L_T}\cos(2\pi\Phi/\Phi_0^* + e^*VL/\hbar v_{\rm p}), \\
I^{\text{I-2}}_{\rm D_3} &= -\frac{8\pi(2\pi)^{2\nu}\alpha}{\nu\Gamma(\nu)^2}\gamma_1^2\gamma_2\gamma_3(e^*V)^{2\nu-2}(k_{\rm B}T)^{2\nu-1}e^{-2L/L_T}\sin^2\pi\nu\cos(2\pi\Phi/\Phi_0^*), \\
I^{\rm II}_{\rm D_3} &= \frac{4(2\pi)^{2\nu}\pi\alpha\csc(\pi\nu)}{\Gamma(2\nu)}\gamma_1^2\gamma_2\gamma_3(e^*V)^{2\nu-1}(k_{\rm B}T)^{2\nu-1}\sqrt{1+h_1(T/V)}\frac{2L+CL_T}{\hbar v_{\rm p}} \\
&\quad\times e^{-2L/L_T}\sin^2\pi\nu\cos(2\pi\Phi/\Phi_0^* + \pi\nu + h_2(T/V)), \\
I^{\rm III}_{\rm D_3} &= \frac{4(2\pi)^{2\nu}\pi\alpha\csc(\pi\nu)}{\Gamma(2\nu)}\gamma_1^2\gamma_2\gamma_3(e^*V)^{2\nu-1}(k_{\rm B}T)^{2\nu-1}\sqrt{1+h_1(T/V)}\frac{CL_T}{\hbar v_{\rm p}} \\
&\quad\times e^{-2L/L_T}\sin^2\pi\nu\cos(2\pi\Phi/\Phi_0^* - \pi\nu - h_2(T/V)),
\end{aligned}$$

where $C \simeq \nu[\psi(\nu)-\psi(1)]/2$, $\alpha \equiv (e^*/\hbar)a^{4\nu}/[(\hbar v_{\rm p})^{4\nu}(2\pi a)^4]$, and a is the short-length cutoff; $C = 0.43$ for $\nu = 1/3$ and $C \to 0.5$ as $\nu \to 0$. Note that $f(t) \propto k_{\rm B}T/\omega$ in Supplementary Equation 7 leads to the higher-order corrections of $h_1(T/V) \equiv \left(\frac{2\pi\nu k_{\rm B}T}{e^*V}\right)^2$ and $h_2(T/V) \equiv (1-2\nu)\arctan(\frac{2\pi\nu k_{\rm B}T}{e^*V})$. These corrections are ignored in Equations (3) and (4) of the main text, considering the limit $e^*V \gg k_{\rm B}T$, while they are included in Equation (6) of the main text for better comparison with the numerical result in Figure 3 of the main text.

Supplementary Note 4 : Reference signal. In the main text, we suggest to measure a reference interference signal $I^{\rm int}_{\rm Ref,\,D_3}$, by applying infinitesimal voltage $V_{\rm ref}/2$ at source S_2 and $-V_{\rm ref}/2$ at S_3, but turning off the voltage V at S_1. It is obtained [3] up to the lowest order in γ's,

$$I^{\rm int}_{\rm Ref,\,D_3} \simeq \frac{e^*}{\hbar}\frac{\gamma_2\gamma_3\,a^{2\nu}}{(\hbar v_{\rm p})^{2\nu}(2\pi a)^2}e^*V_{\rm ref}(k_{\rm B}T)^{2\nu-2}e^{-2L/L_T}\cos\frac{2\pi\Phi}{\Phi_0^*} \qquad \text{for } k_{\rm B}T \gg e^*V_{\rm ref}. \qquad (8)$$

Since the reference signal $I^{\rm int}_{\rm Ref,\,D_3}$ and the main interference signal $I^{\rm int}_{\rm D_3}$ are obtained from the same setup under the same external parameters (temperature, gate voltage, magnetic field, etc), the possible side effects affecting the main signal, including the external-parameter dependence of the size, shape, QPC tunneling, and anyon excitations of the interferometry, appear in the reference in the same manner. Therefore, by comparing the reference with the main signal, one can exclude the side effects and unambiguously detect the interference phase shift θ in Eq. (6), hence, the fractional statistics. This strategy works well both in the pure Aharonov-Bohm regime and the Coulomb-dominated regime.

Supplementary Note 5 : Coulomb dominated regime. We discuss about the Coulomb interaction between the edge and the bulk of the interferometry. There are two regimes of Fabry-Perot interferometry, the pure Aharonov-Bohm regime and the Coulomb dominated regime. In the former regime, the interaction is negligible, while it is crucial in the latter [4]. The Fabry-Perot interferometers of recent experiments [5–9] in the fractional quantum Hall regime are in the Coulomb dominated regime. We below compute the interference current $I^{\rm int}_{\rm D_3}$ in the presence of the Coulomb interaction, and show that Equation (6) of the main text is applicable to both of the pure Aharonov-Bohm regime and the Coulomb dominated regime. We then generalize this to the quantum Hall regime of filling factor $\nu' = \nu + \nu_0$ where $\nu = 1/(2n+1)$ and ν_0 is a nonzero integer.

We compute $I^{\rm int}_{\rm D_3}$, combining our chiral Luttinger liquid theory with the capacitive interaction model [4] that successfully describes the Coulomb dominated regime. The interferometer Hamiltonian $H = \sum_i H_{{\rm edge},i} + H_{\rm tun}$ is modified by the Coulomb interaction as

$$\begin{aligned} H \to H + U_{\rm int} Q_{\rm bulk}\Big(\int_d^{d+L} dx :\partial_x\phi_2(x): + \int_0^L dx :\partial_x\phi_3(x):\Big) + U_{\rm bulk} Q_{\rm bulk}^2 \\ = \sum_{i=1,2,3} \frac{\hbar v_{\rm p}}{4\pi\nu}\int_{-\infty}^{\infty} dx : (\partial_x \bar{\phi}_i(x))^2 + H_{\rm bulk} + H_{\rm tun}. \end{aligned} \quad (9)$$

Here, $Q_{\rm bulk} = \nu B A_{\rm area}/\Phi_0 + \nu N_{\rm L} - \bar{q}$ is the number of the net charges localized within the interferometer bulk (inside the interference loop), $A_{\rm area}$ is the area of the interferometer, $N_{\rm L}$ is the net number of quasiparticles minus quasiholes, and $\bar{q}$ is the number of positive background charges induced by the gate voltage applied to the interferometer. $U_{\rm int}$ is the strength of Coulomb interaction between the charges of the interferometer edge and the charges localized in the interferometer bulk, and $U_{\rm bulk}$ is the strength of interaction between the bulk charges. In the second equality of Supplementary Equation 9, we introduce a boson field $\bar{\phi}_i$ for each edge i, $\bar{\phi}_i(x) = \phi_i(x) + \frac{2\pi\nu}{\hbar v_{\rm p}} U_{\rm int} Q_{\rm bulk} \int_{-\infty}^{x} K_i(x')dx'$, where $K_2(x) = 1$ for $d < x < d + L$, $K_3(x) = 1$ for $0 < x < L$, and $K_i(x) = 0$ otherwise. The second term of $\bar{\phi}$ describes the charges $-\frac{2\pi\nu}{\hbar v_{\rm p}} U_{\rm int} Q_{\rm bulk}$ induced per unit length by the interaction. In Supplementary Equation 9, the Hamiltonian is quadratic in $\bar{\phi}_i$ and has $H_{\rm bulk} = (U_{\rm bulk} - \frac{2\pi\nu L}{\hbar v_{\rm p}} U_{\rm int}^2) Q_{\rm bulk}^2$. Note that $U_{\rm bulk} - \frac{2\pi\nu L}{\hbar v_{\rm p}} U_{\rm int}^2 > 0$. The Keldysh Green's function of $\bar{\phi}_i$ is identical to that of ϕ_i in Supplementary Equation 2.

Repeating the calculation for the case of $U_{\rm int} = U_{\rm bulk} = 0$ and $N_{\rm L} = 0$, we compute the main interference signal $I^{\rm int}_{\rm D_3}$ and the reference signal $I^{\rm int}_{\rm Ref,\, D_3}$. The reference signal is written as

$$\langle I^{\rm int}_{\rm Ref,\, D_3} \rangle \propto {\rm Re}\left[\frac{\sum_{N_{\rm L}=-\infty}^{\infty} e^{-\beta H_{\rm bulk}} \exp(-i2\pi(\nu B A_{\rm area}/\Phi_0 + \nu N_{\rm L}) + i2\pi \frac{\nu U_{\rm int} 2L}{\hbar v_{\rm p}} Q_{\rm bulk})}{\sum_{N_{\rm L}=-\infty}^{\infty} e^{-\beta H_{\rm bulk}}} \right]. \quad (10)$$

Here, $\exp(-i2\pi\nu N_{\rm L})$ counts the phase $2\pi\nu N_{\rm L}$ by the braiding of an interfering anyon around the anyons of the interferometer bulk, and $\sum_{N_{\rm L}} \cdots$ is for the ensemble average over thermal fluctuations of $N_{\rm L}$. Substituting $Q_{\rm bulk} = \nu B A_{\rm area}/\Phi_0 + \nu N_{\rm L} - \bar{q}$, we obtain

$$\langle I^{\rm int}_{\rm Ref,\, D_3} \rangle \propto e^{\frac{-\pi^2\left(1-\frac{2\nu L}{\hbar v_{\rm p}} U_{\rm int}\right)^2}{\beta(U_{\rm bulk} - \frac{2\pi\nu L}{\hbar v_{\rm p}} U_{\rm int}^2)}} \frac{{\rm Re}\left[e^{-i2\pi\bar{q}} \Theta_3\Big(\frac{\pi B A_{\rm area}}{\Phi_0} - \frac{\pi\bar{q}}{\nu} + \frac{i\pi^2\left(1-\frac{2\nu L}{\hbar v_{\rm p}}U_{\rm int}\right)}{\beta\nu\left(U_{\rm bulk} - \frac{2\pi\nu L}{\hbar v_{\rm p}} U_{\rm int}^2\right)}, \exp\big(\frac{-\pi^2\beta^{-1}\nu^{-2}}{(U_{\rm bulk} - \frac{2\pi\nu L}{\hbar v_{\rm p}} U_{\rm int}^2)}\big)\Big)\right]}{\Theta_3\Big(\frac{\pi B A_{\rm area}}{\Phi_0} - \frac{\pi \bar{q}}{\nu}, \exp\big(\frac{-\pi^2\beta^{-1}\nu^{-2}}{(U_{\rm bulk} - \frac{2\pi\nu L}{\hbar v_{\rm p}} U_{\rm int})}\big)\Big)}. \quad (11)$$

$\Theta_3(z, q) = 1 + 2\sum_{n=1}^{\infty} q^{n^2} \cos(2nz)$ is the Jacobi theta function and obeys $\Theta_3(z, q) = \Theta_3(z + \pi, q)$. In the Coulomb dominated regime of $\hbar v_{\rm p}/(2\nu L) \simeq U_{\rm int}$, $I^{\rm int}_{\rm Ref,\, D_3}$ is independent of the magnetic field B, as Θ_3's in the numerator and the denominator cancel each other. In the pure Aharonov-Bohm regime, $I^{\rm int}_{\rm Ref,\, D_3}$ is a periodic function of B with periodicity $\Phi_0/A_{\rm area}$.

On the other hand, $I^{\rm int}_{\rm Ref,\, D_3}$ shows periodic oscillations as a function of the gate voltage in both the regimes, since $\bar{q}$ and $A_{\rm area}$ depend on the gate voltage. To see this, we write $I^{\rm int}_{\rm Ref,\, D_3}$ as

$$\langle I^{\rm int}_{\rm Ref,\, D_3} \rangle \propto \frac{{\rm Re}\left[\Theta_3\Big(\big(\pi\bar{\alpha} - \frac{\pi\bar{\gamma}}{\nu}\big) V_{\rm G} + \frac{i\pi^2\left(1-\frac{2\nu L}{\hbar v_{\rm p}}U_{\rm int}\right)}{\beta\nu\left(U_{\rm bulk} - \frac{2\pi\nu L}{\hbar v_{\rm p}}U_{\rm int}^2\right)}, \exp\big(\frac{-\pi^2\beta^{-1}\nu^{-2}}{(U_{\rm bulk} - \frac{2\pi\nu L}{\hbar v_{\rm p}}U_{\rm int}^2)}\big)\Big) e^{-i2\pi\bar{\gamma}V_{\rm G}} \right]}{\Theta_3\Big(\big(\pi\bar{\alpha} - \frac{\pi\bar{\gamma}}{\nu}\big) V_{\rm G}, \exp\big(-\frac{\pi^2\beta^{-1}\nu^{-2}}{(U_{\rm bulk} - \frac{2\pi\nu L}{\hbar v_{\rm p}}U_{\rm int})}\big)\Big)}, \quad (12)$$

where $\bar{\gamma} \equiv \frac{d\bar{q}}{dV_{\rm G}}$ and $\bar{\alpha} \equiv \frac{B}{\Phi_0}\frac{dA_{\rm area}}{dV_{\rm G}}$. The oscillation period is $1/\bar{\gamma}$ $(= V_{\rm G,0})$ in the Coulomb dominated regime of $U_{\rm int} \simeq \hbar v_{\rm p}/(2\nu L)$ and also in the pure Aharonov-Bohm regime of $U_{\rm int} \simeq 0$ and $\beta U_{\rm bulk} \ll 1$. All these results are in good agreement with the previous results in [4].

And we obtain the main interference signal $I^{\rm int}_{\rm D_3}$, combining Supplementary Equation 3 and the ensemble average over thermal fluctuations of $N_{\rm L}$ which is considered in Supplementary Equation 10,

$$\langle I^{\rm int}_{\rm D_3}\rangle \propto \frac{{\rm Re}\left[e^{-i2\pi\bar{q}}e^{-i\theta}\Theta_3\left(\frac{\pi B A_{\rm area}}{\Phi_0}-\frac{\pi\bar{q}}{\nu}+\frac{i\pi^2\left(1-\frac{2\nu L}{\hbar v_{\rm p}}U_{\rm int}\right)}{\beta\nu\left(U_{\rm bulk}-\frac{2\pi\nu L}{\hbar v_{\rm p}}U^2_{\rm int}\right)},\exp\left(\frac{-\pi^2\beta^{-1}\nu^{-2}}{(U_{\rm bulk}-\frac{2\pi\nu L}{\hbar v_{\rm p}}U^2_{\rm int})}\right)\right)\right]}{\Theta_3\left(\pi\frac{\pi B A_{\rm area}}{\Phi_0}-\frac{\pi\bar{q}}{\nu},\exp\left(-\frac{\pi^2\beta^{-1}\nu^{-2}}{(U_{\rm bulk}-\frac{2\pi\nu L}{\hbar v_{\rm p}}U_{\rm int})}\right)\right)}, \tag{13}$$

Notice that Supplementary Equation 13 is identical to Supplementary Equation 11 except $e^{-i\theta}$. We find that the phase shift θ in Supplementary Equation 13 satisfies the expression in Supplementary Equation 4, hence, follows Equation (6) (see the main text) in the semiclassical regime of $e^*V \gg k_{\rm B}T \gtrsim \hbar v_{\rm p}/L$. Supplementary Equations 11, 12, and 13 are used for drawing Fig. 3.

The phase θ results from the topological vacuum bubbles. θ is detectable both in the Coulomb dominated regime and the pure Aharonov-Bohm regime. In the Coulomb dominated regime of $U_{\rm int}\simeq \hbar v_{\rm p}/(2\nu L)$, $I^{\rm int}_{\rm D_3}\propto {\rm Re}[e^{-i2\pi\bar{\gamma}V_{\rm G}}e^{-i\theta}]$ and $I^{\rm int}_{\rm Ref,\ D_3}\propto {\rm Re}[e^{-i2\pi\bar{\gamma}V_{\rm G}}]$ are independent of the magnetic field. In this case, the interference pattern of $I^{\rm int}_{\rm D_3}$ is shifted by θ from that of $I^{\rm int}_{\rm Ref,\ D_3}$ as the gate voltage $V_{\rm G}$ varies. On the other hand, in the pure Aharonov-Bohm regime of $U_{\rm int}\simeq 0$ and when $\beta U_{\rm bulk}\ll 1$, $I^{\rm int}_{\rm D_3}\propto {\rm Re}[e^{-i\theta}e^{-i(2\pi BA_{\rm area}/\Phi_0-2\pi\bar{q}/\nu)}]$ and $I^{\rm int}_{\rm Ref,\ D_3}\propto {\rm Re}[e^{-i(2\pi BA_{\rm area}/\Phi_0-2\pi\bar{q}/\nu)}]$. Note that the period of the magnetic-field dependence is determined by $\Phi_0\equiv h/e$, rather than the anyon flux quantum $\Phi^*_0 = h/e^*$, because the change of $N_{\rm L}$ (the net number of anyonic quasiparticles minus anyonic quasiholes localized inside the interferometry loop) is allowed in the model of Supplementary Equation 9; see Ref. [3, 10]. In this case, the interference pattern of $I^{\rm int}_{\rm D_3}$ is shifted by θ from that of $I^{\rm int}_{\rm Ref,\ D_3}$, as either the magnetic field or the gate voltage varies.

The Coulomb interaction parameters chosen for the left panel of Fig. 3a are $U_{\rm bulk} = 10\hbar v_{\rm p}/(2\nu L)$ and $U_{\rm int} = 0.1\hbar v_{\rm p}/(2\nu L)$, while $U_{\rm bulk} = 10\hbar v_{\rm p}/(2\nu L)$ and $U_{\rm int} = 0.9\hbar v_{\rm p}/(2\nu L)$ for the right panel.

Next, we consider a more general quantum Hall regime of filling factor $\nu' = \nu+\nu_0$ where $\nu = 1/(2n+1)$ and ν_0 is a nonzero integer, focusing on the situation that the edge channels from the integer filling ν_0 are fully transmitted through the QPCs while those from the fractional filling ν participate in the Fabry-Perot interference as in Fig 2. As shown below, the presence of the edge channels from the integer filling ν_0 does not modify our main finding. Namely, in this case the interference-pattern phase shift between $I^{\rm int}_{\rm Ref,\ D_3}$ and $I^{\rm int}_{\rm D_3}$ is identical to the phase θ of the $\nu_0 = 0$ case discussed in Equation (6) of the main text.

The Hamiltonian of this more general situation is the same as that in Supplementary Equation 9 but with the replacement of $Q_{\rm bulk} = \nu BA_{\rm area}/\Phi_0+\nu N_{\rm L}-\bar{q}$ into $Q_{\rm bulk} = \nu' BA_{\rm area}/\Phi_0+\nu N_{\rm L}-\bar{q}$. The replacement $\nu\to\nu' = \nu+\nu_0$ counts the fact that the total amount of electron changes occupying the Landau levels of the interferometer bulk region is now $\nu' BA_{\rm area}/\Phi_0$. In the same way as before (see Supplementary Equation 10), we obtain the reference signal

$$\langle I^{\rm int}_{\rm Ref,\ D_3}\rangle \propto e^{\frac{-\pi^2\left(1-\frac{2\nu L}{\hbar v_{\rm p}}U_{\rm int}\right)^2}{\beta(U_{\rm bulk}-\frac{2\pi\nu L}{\hbar v_{\rm p}}U^2_{\rm int})}}\frac{{\rm Re}\left[e^{i2\pi(\frac{\nu_0 BA_{\rm area}}{\Phi_0}-\bar{q})}\Theta_3\left(\frac{\nu'\pi BA_{\rm area}}{\nu\Phi_0}-\frac{\pi\bar{q}}{\nu}+\frac{i\pi^2\left(1-\frac{2\nu L}{\hbar v_{\rm p}}U_{\rm int}\right)}{\beta\nu\left(U_{\rm bulk}-\frac{2\pi\nu L}{\hbar v_{\rm p}}U^2_{\rm int}\right)},\exp\left(\frac{-\pi^2\beta^{-1}\nu^{-2}}{(U_{\rm bulk}-\frac{2\pi\nu L}{\hbar v_{\rm p}}U^2_{\rm int})}\right)\right)\right]}{\Theta_3\left(\frac{\nu'\pi BA_{\rm area}}{\nu\Phi_0}-\frac{\pi\bar{q}}{\nu},\exp\left(\frac{-\pi^2\beta^{-1}\nu^{-2}}{(U_{\rm bulk}-\frac{2\pi\nu L}{\hbar v_{\rm p}}U_{\rm int})}\right)\right)}. \tag{14}$$

We first discuss the magnetic-field dependence of the reference signal. In the Coulomb dominated regime of $U_{\rm int}\simeq \hbar v_{\rm p}/(2\nu L)$, the reference signal becomes $I^{\rm int}_{\rm Ref,\ D_3}\propto {\rm Re}[e^{2\pi i(\nu_0 BA_{\rm area}/\Phi_0-\bar{q})}]$, showing magnetic-field dependent oscillations with the period determined by ν_0. This result coincides with an expression obtained with a capacitive interaction model in Ref. [4]; cf. Eqs. (24) and (25) of Ref. [4]. In the pure Aharonov-Bohm regime of $U_{\rm int}\simeq 0$ and when $\beta U_{\rm bulk}\ll 1$, the reference signal becomes $I^{\rm int}_{\rm Ref,\ D_3}\propto {\rm Re}[e^{2\pi i(\nu_0 BA_{\rm area}/\Phi_0-\bar{q})}(1+\frac{1}{2}\exp(\frac{\pi^2(2\nu-1)}{\beta\nu^2 U_{\rm bulk}})e^{-2\pi i(\nu' BA_{\rm area}/(\nu\Phi_0)-\bar{q}/\nu)})]$. This also agrees with Ref. [4]; cf. the two dominant terms of Eq. (24) of Ref. [4]. For the integer quantum Hall regime of $(\nu',\nu_0) = (2,1)$, this expression becomes $I^{\rm int}_{\rm Ref,\ D_3}\propto {\rm Re}[e^{-2\pi iBA_{\rm area}/\Phi_0}]$, which agrees with the experimental data in Ref. [8] for Aharonov-Bohm oscillations in the Fabry-Perot interferometry in the bulk filling factor $\nu' = 2$ and with one fully transmitting channel ($\nu_0 = 1$). Next, we discuss the gate-voltage dependence of the reference signal. The gate-voltage dependent part of the reference signal becomes $I^{\rm int}_{\rm Ref,\ D_3}\propto {\rm Re}[e^{-i2\pi(\bar{\gamma}-\nu_0\bar{\alpha})V_{\rm G}}]$ in the Coulomb dominated regime of $U_{\rm int}\simeq \hbar v_{\rm p}/(2\nu L)$, and $I^{\rm int}_{\rm Ref,\ D_3}\propto {\rm Re}[e^{-2\pi i(\bar{\gamma}-\nu_0\bar{\alpha})V_{\rm G}}(1+\frac{1}{2}\exp(\frac{\pi^2(2\nu-1)}{\beta\nu^2 U_{\rm bulk}})e^{-2\pi i(\nu'\bar{\alpha}V_{\rm G}/\nu-\bar{\gamma}V_{\rm G}/\nu)})]$ in the pure Aharonov-Bohm regime of $U_{\rm int}\simeq 0$ and $\beta U_{\rm bulk}\ll 1$. The gate-voltage dependences are again in good agreement with Ref. [4].

We also obtain the main interference signal $I^{\rm int}_{\rm D_3}$ for the general case of $\nu' = \nu + \nu_0$,

$$\langle I^{\rm int}_{\rm D_3}\rangle \propto \frac{\mathrm{Re}\left[e^{i2\pi(\frac{\nu_0 B A_{\rm area}}{\Phi_0}-\bar{q})} e^{-i\theta} \Theta_3\left(\frac{\nu'\pi B A_{\rm area}}{\nu\Phi_0} - \frac{\pi\bar{q}}{\nu} + \frac{i\pi^2\left(1-\frac{2\nu L}{\hbar v_{\rm p}}U_{\rm int}\right)}{\beta\nu\left(U_{\rm bulk}-\frac{2\pi\nu L}{\hbar v_{\rm p}}U^2_{\rm int}\right)}, \exp\left(\frac{-\pi^2\beta^{-1}\nu^{-2}}{(U_{\rm bulk}-\frac{2\pi\nu L}{\hbar v_{\rm p}}U^2_{\rm int})}\right)\right)\right]}{\Theta_3\left(\frac{\nu'\pi B A_{\rm area}}{\nu\Phi_0} - \frac{\pi\bar{q}}{\nu}, \exp\left(\frac{-\pi^2\beta^{-1}\nu^{-2}}{(U_{\rm bulk}-\frac{2\pi\nu L}{\hbar v_{\rm p}}U_{\rm int})}\right)\right)}. \quad (15)$$

Again as in the previous case of $\nu_0 = 0$, Supplementary Equation 15 is identical to Supplementary Equation 14 except $e^{-i\theta}$, and the phase shift θ satisfies the expression in Eq. (6) (see the main text) in the semiclassical regime of $e^*V \gg k_{\rm B}T \gtrsim \hbar v_{\rm p}/L$. Therefore, θ is detectable also in the general case of $\nu' = \nu + \nu_0$ from the phase shift between the reference signal and the main signal.

Supplementary Note 6 : Discussion about Eqs. (3) and (4). We discuss Equations (3) and (4) of the main text, focusing on the physical interpretation of Type II which involves topological vacuum bubbles. We repeat the expression for Type II in Eq. (3),

$$I^{\rm II}_{\rm D_3} \;\propto\; \gamma_1^2\gamma_2\gamma_3(e^*V)^{2\nu-1}(k_{\rm B}T)^{2\nu-1}\frac{2L+CL_T}{\hbar v_{\rm p}}e^{-2L/L_T}\sin^2\pi\nu\cos(2\pi\Phi/\Phi_0^*+\pi\nu),$$

The factors $(e^*V)^{2\nu}$ and $(k_{\rm B}T)^{2\nu}$ arise from the tunneling of an anyon biased by e^*V and that of a thermally excited anyon, respectively. Other factors $(e^*V)^{-1}$, $(k_{\rm B}T)^{-1}$, and e^{-2L/L_T} describe the reduction of wave packet overlap, hence the reduction of interference amplitude. In particular the last one, e^{-2L/L_T}, is the well-known dephasing factor. The factor $2L + CL_T$ comes from the phase space for anyon braiding of Type II. In the factor of $\sin^2\pi\nu$, one power, $\sin\pi\nu$, originates from the braiding phase $2\pi\nu$ and the vacuum bubble effect (see Supplementary Figure 2 and the next paragraph), while the other $\sin\pi\nu$ originates from the exchange between two thermally excited anyons. The other expressions of Types I-1, I-2, and III in Eq. (4) can be understood in a similar way.

We below explain how the braiding phase $2\pi\nu$ effectively occurs in Type II process.

- The two interfering paths of a Type II process are shown in Supplementary Figures 2a and b.
- A gauge describing the exchange-statistics phase of anyons between different edges (Edges 2 and 3) is chosen for tunneling operators at QPC2 and QPC3, as shown in Equation (12) of the main text. We choose the gauge in parallel to the extended edge scheme in Fig. 4 of the main text.
- When the particle-like "virtual" anyon thermally excited on Edge 2 at QPC2 jumps to Edge 3 (see Supplementary Figure 2a), it gains the exchange-statistics phase factor $\zeta_a = e^{-i\pi\nu}$ as its location is exchanged with the voltage-biased "real" anyon injected from Edge 1; see Supplementary Figure 2c. In the same gauge, the particle-like "virtual" anyon thermally excited on Edge 2 at QPC3 in Supplementary Figure 2b gains no exchange-statistics phase factor, when it jumps to Edge 3.
- After the jump, the spatial ordering of anyons on Edge 2 is different between Supplementary Figures 2a and b. For the paths 2a and b to interfere, the anyons (the particle-like real anyon and the hole-like virtual anyon) for example in Supplementary Figure 2b must be exchanged as in Supplementary Figure 2d, for them to have the same spatial ordering as Supplementary Figure 2a. This results in another exchange-statistics phase factor of $\zeta_b = e^{i\pi\nu}$ shown in Supplementary Figure 2d.
- The two factors lead to the winding phase factor $\zeta_a^*\zeta_b = e^{i2\pi\nu}$ in the interference signal.
- The phase factors ζ_a and ζ_b are derived from the commutation rules in Eqs. (11) and (12).

We further discuss Eqs. (3) and (4). The phase shift $e^*VL/\hbar v_{\rm p}$ in $I^{\rm I\text{-}1}_{\rm D_3} \propto \cos(2\pi\Phi/\Phi_0^* + e^*VL/\hbar v_{\rm p})$ originates from the average of dynamical phase $2kL$ over the energy window of e^*V, where $\hbar k$ is anyon momentum. $I^{\rm I\text{-}2}_{\rm D_3}$ is proportional to $\cos(2\pi\Phi/\Phi_0^*)$ without the shift $\propto e^*V$. It is because Type I-2 is the interference between the anyon of energy e^*V injected from S_1 and a thermally excited anyon so that the energy window for the average of the dynamical phase is determined by $\min\{e^*V, k_{\rm B}T\} = k_{\rm B}T$. $I^{\rm II}_{\rm D_3} \propto \cos(2\pi\Phi/\Phi_0^* + \pi\nu)$ and $I^{\rm III}_{\rm D_3} \propto \cos(2\pi\Phi/\Phi_0^* - \pi\nu)$ do not have the phase shift $\propto e^*V$, because the interference occurs by thermally excited anyons.

We point out that the phase shift θ is well described by Eq. (6) (see the main text) in the regime of $e^*V \gg k_{\rm B}T, \hbar v_{\rm p}/L$ (beyond $e^*V \gg k_{\rm B}T \gg \hbar v_{\rm p}/L$). It is because Types II and III share the common physics except only for the phase space ($2L + CL_T$ and CL_T, respectively) for anyon braiding.

Supplementary References

[1] von Delft, J. & Schoeller, H., Bosonization for Beginners—Refermionization for Experts. *Ann, Phys.* (Leipzig) **7,** 225-306 (1998).

[2] Kim, E.-A., Lawler, M., Vishveshwara, S. & Fradkin, E. Measuring fractional charge and statistics in fractional quantum Hall fluids through noise experiments. *Phys. Rev. B* **74,** 155324 (2006).

[3] Chamon, C. D. C., Freed, D. E., Kivelson, S. A., Sondhi, S. L. & Wen, X. G. Two point-contact interferometer for quantum Hall systems. *Phys. Rev. B* **55,** 2331-2343 (1997).

[4] Halperin, B., Stern, A., Neder, I. & Rosenow, B. Theory of the Fabry-Perot quantum Hall interferometer, *Phys. Rev. B* **83,** 155440 (2011).

[5] An, S. *et al.* Braiding of Abelian and Non-Abelian Anyons in the Fractional Quantum Hall Effect. preprint at http://arXiv.org/abs/1112.3400 (2011).

[6] Camino, F. E., Zhou, W. & Goldman, V. J. e/3 Laughlin Quasiparticle Primary-Filling =1/3 Interferometer. *Phys. Rev. Lett.* **98,** 076805 (2007).

[7] Willett, R. L., Pfeiffer, L. N. & West, K. W. Measurement of filling factor 5/2 quasiparticle interference with observation of charge e/4 and e/2 period oscillations. *Proc. Natl. Acad. Sci. U.S.A* **106,** 8853-8858 (2009).

[8] Ofek, N. *et al.* Role of interactions in an electronic Fabry Perot interferometer operating in the quantum Hall effect regime. *Proc. Natl. Acad. Sci. U.S.A* **107,** 5276-5281 (2010).

[9] McClure, D. T., Chang, W., Marcus, C. M., Pfeiffer, L. N. & West, K. W. Fabry-Perot Interferometry with Fractional Charges. *Phys. Rev. Lett.* **108,** 256804 (2012).

[10] Kivelson, S. Semiclassical theory of localized many-anyon states. *Phys. Rev. Lett.* **65,** 3369-3372 (1990).
